# Impact of Maternal Lifestyle and Dietary Habits during Pregnancy on Newborn Metabolic Profile

**DOI:** 10.3390/nu15102297

**Published:** 2023-05-13

**Authors:** Ilaria Cicalini, Samanta Moffa, Maria Lucia Tommolini, Silvia Valentinuzzi, Mirco Zucchelli, Ines Bucci, Piero Chiacchiaretta, Antonella Fontana, Luca Federici, Vincenzo De Laurenzi, Piero Del Boccio, Claudia Rossi, Damiana Pieragostino

**Affiliations:** 1 Center for Advanced Studies and Technology (CAST), “G. d’Annunzio” University of Chieti-Pescara, 66100 Chieti, Italy; ilaria.cicalini@unich.it (I.C.); maria.tommolini@unich.it (M.L.T.); silvia.valentinuzzi@unich.it (S.V.); m.zucchelli@unich.it (M.Z.); ines.bucci@unich.it (I.B.); p.chiacchiaretta@unich.it (P.C.); luca.federici@unich.it (L.F.); vincenzo.delaurenzi@unich.it (V.D.L.); p.delboccio@unich.it (P.D.B.); damiana.pieragostino@unich.it (D.P.); 2 Department of Innovative Technologies in Medicine and Dentistry, “G. d’Annunzio” University of Chieti-Pescara, 66100 Chieti, Italy; 3 Department of Pharmacy, “G. d’Annunzio” University of Chieti-Pescara, 66100 Chieti, Italy; samanta.moffa@unich.it (S.M.); antonella.fontana@unich.it (A.F.); 4 Department of Medicine and Aging Science, “G. d’Annunzio” University of Chieti-Pescara, 66100 Chieti, Italy

**Keywords:** newborn screening, metabolic profiling, mass spectrometry, dietary habits, lifestyle, nutrition

## Abstract

Expanded newborn screening (NBS) is a preventive program that allows for the early identification of over 40 congenital endocrine-metabolic diseases by analyzing dried blood spot samples collected from the newborn’s heel within 48–72 h of birth. The determination of amino acids and acyl-carnitines by Flow Injection Analysis Tandem Mass Spectrometry (FIA-MS/MS) may also highlight metabolic alterations resulting from external factors, such as maternal nutrition. In the present study, we developed a questionnaire to investigate the eating habits of 109 women during pregnancy and statistically correlated the results from the investigation on dietary habits with the data obtained by the NBS laboratory of Abruzzo region (Italy). Parameters such as smoking, physical activity, and the intake of iodized salt, drugs, and supplements were analyzed. This study aimed to highlight how maternal lifestyle, diet, and drug intake during pregnancy may affect the neonatal metabolic profile, possibly generating false positive or false negative results in the NBS test. The results pointed out how the knowledge of maternal nutrition and lifestyle may also be precious in preventing misinterpretations of the neonatal metabolic profile, thereby reducing unnecessary stress for newborns and their parents and limiting costs for the health system.

## 1. Introduction

Expanded Newborn Screening (NBS) is a preventive medicine program whose goal is to identify infants at risk for over 40 inherited endocrine-metabolic diseases caused by specific enzymatic defects, which determine the accumulation of toxic metabolic intermediates and which, if not detected promptly at birth, can compromise the patient’s life with severe and often irreversible damage. The analysis is performed on dried blood spot (DBS) samples obtained by pricking the newborn’s heel generally within 48–72 h of birth and by collecting and letting dry the drops of whole blood on a special filter paper. To date, the Italian panel of diseases object of the expanded NBS includes congenital hypothyroidism (CH), cystic fibrosis (CF), galactosemia, biotinidase deficiency (BTD), aminoacidopathies (AA), urea cycle disorders (UCD), organic aciduria (OA) and fatty acid oxidation disorders (FAOD), for a total of over than 40 inborn errors of metabolism (IEMs). The advantage offered by the NBS in terms of time is guaranteed by the use of tandem mass spectrometry (MS/MS), a versatile, specific, and sensitive technology that is becoming essential for high-throughput analysis. In fact, in a single analytical run of approximately 1 min, it provides a semi-quantitative picture of different metabolites (amino acids, acyl-carnitines, succinyl-acetone) as markers of the diseases of interest [1,2]. Moreover, this targeted metabolomic approach ensures excellent analytical precision and facilitates the identification and quantification of metabolites [3]. In particular, the MS-based NBS strategy allows the diagnostic process to be undertaken early and to promptly start the necessary treatments, thus improving the child’s health and changing the natural history of the disease. It is worth remembering that various maternal and neonatal factors can affect the interpretation of NBS results. Interestingly, there is ample evidence that the metabolic results of the NBS test can be influenced by maternal lifestyle, nutrition, pharmacological treatments, and diseases, as well as neonatal clinical variables such as prematurity, the type of feeding, parenteral nutrition, sepsis, and distress [4,5,6]. In these cases, the metabolic alterations at expanded NBS could be a transitory condition generating false positive results. The most consistent false positives are associated with the suspect of propionic and methyl-malonic acidemia, where various predisposing factors are considered, such as sepsis, hypoxic-ischemic encephalopathy, drug intoxication due to maternal exposure, and other congenital disorders of metabolism [7]. Often, the altered profile of C3 (propionyl-carnitine), the C3/C2 (C3/acetyl-carnitine) ratio, C16: 1OH\C17 (3-hydroxy-hexadecenoylcarnitine) and methionine is attributable to maternal vitamin B12 deficiencies due to poor diets or being totally free of animal products [4], untreated pernicious anemia or nutrient absorption dysfunctions (e.g., gastric bypass). Vitamin B12 deficiency in pediatric populations can predispose increased plasma homocysteine levels, an emerging risk factor for cardiovascular disease, low bone mineral density predisposing to osteoporosis, neural tube defects, intrauterine growth retardation, autism spectrum disorder (ASD) and attention deficit hyperactivity disorder (ADHD) [8] while in pregnant women there is a risk of premature birth, preeclampsia, and spontaneous abortion [9]. Therefore, it is clear that maternal nutrition and lifestyle can play a crucial role in the growth, health, and well-being of the fetus [10].

The infant’s feeding can, in turn, alter the metabolite profile. In fact, nutritional supports rich in amino acids can cause false positives: total parenteral nutrition is often able to return false alterations to mimic congenital hyperphenylalaninemia [11,12] or other AA.

Another factor that determines the alteration of metabolites at screening may be the intake of drugs during pregnancy and breastfeeding. It is well known that some antibiotics, such as ampicillin and cefotaxime, can induce an increase in the level of acyl-carnitines (C5, C14: 1, and C16: 1-OH) due to the presence of the pivaloyl functional group [3].

Taken together, all these aspects highlight the importance of post-analytical interpretive tools to resolve the issue of false-positive results at newborn screening for IEMs by using the information on clinical variables potentially associated with abnormal outcomes.

On the basis of this evidence, in this study, we realized a questionnaire to investigate the eating habits of 109 women during pregnancy and statistically correlated the results from the investigation with around 80 metabolic parameters, including metabolic ratios, obtained by the NBS laboratory of Abruzzo region, Italy. More precisely, we investigated factors related to lifestyle (physical activity and smoking), dietary habits during pregnancy (including iodized salt consumption), and also the intake of drugs and supplements (progesterone, folic acid) and demonstrated their influence on the metabolic profile of newborns. These factors can eventually overlap with pathological conditions and possibly generate false positives and/or false negatives in NBS results, supporting the importance of an adequate nutritional status during pregnancy. Our approach, through a better knowledge of maternal nutrition and lifestyle, aims at improving our understanding of the neonatal metabolic profile and avoiding misinterpretations, thus allowing a reduction in unnecessary stress for newborns and their families and limiting the costs of the health system.

## 2. Materials and Methods

### 2.1. Routinely Newborn Screening Analysis

Dried blood spot (DBS) samples for NBS were punched out into 3.2 mm disks to perform a flow injection-tandem mass spectrometry analysis (FIA-MS/MS) for the detection of over 40 IEMs, including AAs, urea cycle, organic acid, and fatty acid oxidation disorders. Four 3.2 mm DBS disks were used for immunofluorimetric assays by a Genetic Screening Processor GSP^®^ Instrument (Perkin Elmer Life and Analytical Sciences, Turku, Finland) to test for congenital hypothyroidism (CH), cystic fibrosis (CF), galactosemia and biotinidase deficiency, respectively, and the fifth DBS disk was employed for FIA-MS/MS analysis. For the latter, the DBS disk (of approximately 3–3.2 μL whole blood) was extracted by adding 125 µL of extraction solutions containing an internal standard provided by the NeoBase 2 Non-Derivatized MSMS Kit (Perkin Elmer Life and Analytical Sciences, Turku, Finland) for 30 min at 45 °C at 700 rpm. After the incubation step, 100 µL of the solution was transferred onto a clear plate for the next determination of 14 AAs, 35 acylcarnitines (ACs), free carnitine, and succinylacetone by FIA-MS/MS.

The MS/MS system consists of RenataDX™ Screening Systems (Waters Corporation, Milford, MA, USA) as a fully FIA-MS/MS IVD system for high-throughput analysis. The system components include the 3777C IVD Sample Manager, ACQUITY™ UPLC™ I-Class IVD Binary Solvent Manager, and Xevo™ TQD IVD Mass Spectrometer (Waters Corporation, Milford, MA, USA). The system operates in the positive electrospray ionization mode with multiple reaction monitoring (MRM) acquisitions. A total of 10 μL were injected into the ion source, and the run time was 1.3 min, injection-to-injection, as already described [2]. Data were finally processed by the MassLynx™ (IVD) Software V4.2 with IonLynx™ Application Manager (Waters Corp.). Mass spectrometry parameters, including MS/MS transitions, cone potentials, and collision energies for each metabolite and its relative internal standard, are fully listed in Table S1 of the Supplementary Materials [4,11].

### 2.2. Questionnaire

A questionnaire was created to obtain information about the lifestyle and nutrition of women during pregnancy. Significant data were then collected from the volunteer women in post-partum periods from the 9 Birth Points in the Abruzzo region (Chieti, Vasto, Lanciano, L’Aquila, Sulmona, Sant’Omero, Teramo, Pescara, and Avezzano). In total, 109 women participated in the study. They were contacted by telephone and received the questionnaire via smartphone or email. The mothers of newborns who tested positive for the pathologies covered by the expanded NBS as well as mothers affected by diseases that are the object of NBS were not included in the study.

The aim of the questionnaire was to obtain information about the lifestyle (physical activity and smoking) and nutritional habits followed by women during pregnancy (Table S2). In particular, lifestyle, smoking and alcohol intake, the type of diet followed (Mediterranean; Lacto-ovo-vegetarian; Lacto-vegetarian; Ovo-vegetarian; Lacto-ovo-pesco-vegetarian; Vegan; Other types), and the frequency of consumption of each food class (white and red meat, cooked cured meats, raw cured meats, fish, eggs, fresh and aged cheeses, yogurt or milk or milk substitutes, cereals, legumes, vegetables, fresh and dried fruit, nervine drinks and sweets) were of interest. Moreover, it was also decided to examine the intake of drugs and/or the treatment of particular previous pathologies and dietary support supplements (Vitamin A; Vitamin E; Vitamin K; Vitamin C; Vitamin B12; Other Vitamins of group B; Calcium; Iron; Phosphorus; Magnesium; Omega-3; Probiotics; Antioxidants; Proteins/Amino acids; Vegetable supplements), as well as the feeding modalities of the newborn (breast, artificial, mixed and/or total parenteral nutrition).

The questionnaire, as developed in the electronic format on the Google Forms platform, is available at the following link: https://docs.google.com/forms/d/19fRGZf58IcP4fJS01ei6vGKQprv1VxW33x9K9wUmGeE/edit?usp=drivesdk.

### 2.3. Matrix Preparation

A single Excel matrix was prepared containing the information extrapolated from the cards where the newborn’s blood sample was collected, the responses of the mothers to the questionnaire, and the values of amino acids and acylcarnitines obtained by tandem mass spectrometry. A score was assigned to each answer given by the mothers in the questionnaire, as reported in Table S2 of the Supplementary Materials. Factors from the questionnaire were selected from time to time and were statistically correlated to the metabolite concentrations of each newborn.

### 2.4. Statistical Analysis

A statistical correlation between the concentrations of amino acids and acylcarnitines obtained by MS/MS analysis and the responses of interest given by the mothers in the questionnaire was performed. The correlation analyses were processed by GraphPad Prism (GraphPad software, Inc., Boston, MA, USA) and Metaboanalyst 4.0. Supervised multivariate analysis was performed using Sparse PLS-DA (Sparse Partial Least Squares–Determination Analysis), and univariate analysis was performed using the *t*-test and ANOVA. Correlation analysis was performed on the basis of the questionnaire responses and metabolite levels measured in infants who were clustered according to biochemical criteria (amino acids, urea cycle metabolites, short-, medium-, long-chain acylcarnitines and odd-chain acylcarnitines) using the Python sklearn library available online at https://scikit-learn.org/stable/.

## 3. Results

In our study, among all the data collected, some neonatal metabolic parameters correlated with the maternal factors addressed by the questionnaire. In the following, the results obtained by the correlation analysis of the metabolites significantly modulated in relation to physical activity, the use of iodized salt in the diet, and smoking during pregnancy are described.

### 3.1. Correlation Analysis

#### 3.1.1. Physical Activity

**Figure 1:**
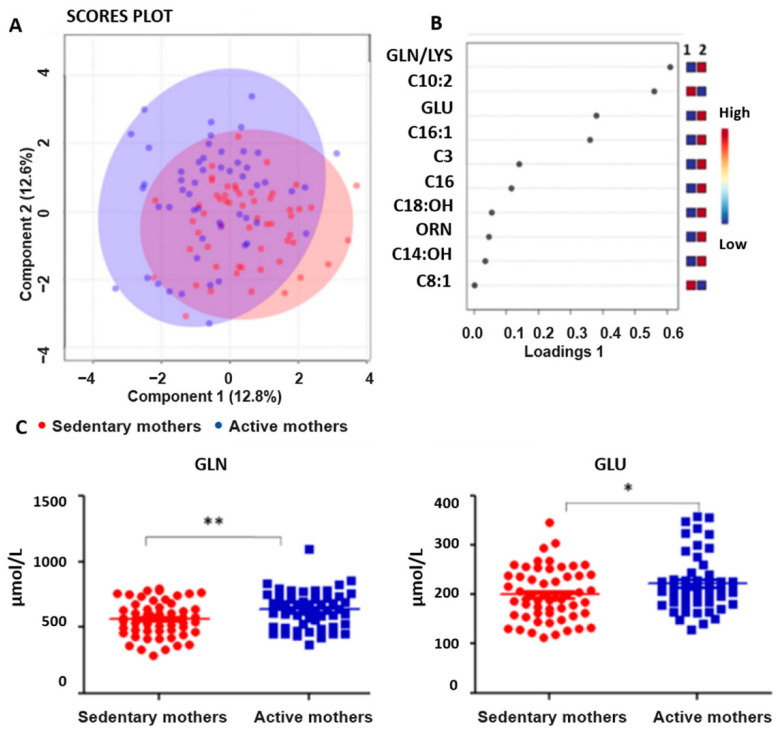
(**A**,**B**) PLS-DA scattered analysis: selection and clustering of variables relating to physical activity: sedentary mothers in red, active mothers in blue. (**C**) Dots plot showing the concentrations in μmol/L for GLN and GLU in newborns with mothers who did not exercise (in red) and who exercised (in blue) during pregnancy (GLN: *p* = 0.0060; GLU: *p* = 0.0302). (*) *p* < 0.05 and (**) *p* < 0.01.

The first factor analyzed was physical activity. The segregation of the total matrix as a function of variables relating to maternal physical activity during pregnancy through sPLS-D statistical analysis shows a scatter plot in which individuals are reported as dots of different colors depending on the amount of physical activity performed. In particular, the red dots (51%) indicate a lack of training, and the blue ones (49%) represent physical activity, as shown in Figure 1A,B.

The univariate statistical analysis by t-test and the elimination of the outliers highlighted a significant difference in the levels of glutamine (GLN) and glutamate (GLU) in the metabolic profile of newborns with active mothers during pregnancy (GLN *p* < 0.001; GLU *p* < 0.01), as represented in Figure 1C. More precisely, in this comparison, a higher concentration of GLN and GLU were observed by the MS/MS screening analysis of DBS samples from newborns with mothers who were physically active during pregnancy.

#### 3.1.2. Use of Iodized Salt in the Diet

**Figure 2:**
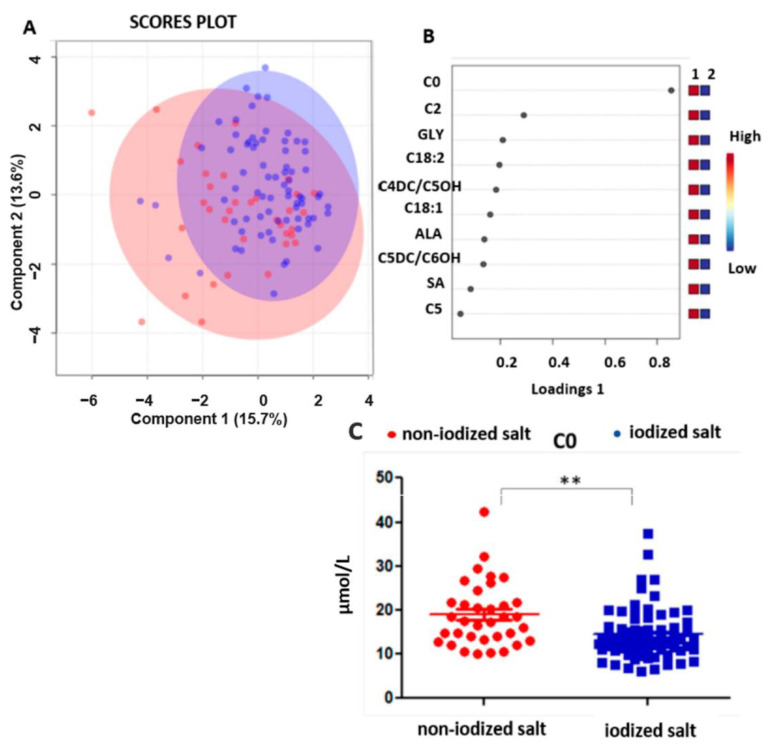
(**A**,**B**) PLS-DA scattered analysis: selection and clustering of variables relating to the use of iodized salt in the diet: maternal intake of non-iodized salt during pregnancy in red, maternal intake of iodized salt during pregnancy in blue. (**C**) Dots plot showing the concentrations in μmol/L for C0 in newborns with maternal non-intake of iodized salt (in red) and maternal intake of iodized salt (in blue) during pregnancy (C0: *p* = 0.00169). (**) *p* < 0.01.

The segregation of the total matrix as a function of the consumption of iodized salt during pregnancy by sPLS-DA statistical analysis produced an undefined separation between the two groups. Figure 2A,B shows the scatter plot with dots of different colors depending on the maternal habit for the consumption of iodized salt during pregnancy.

In particular, the intake of iodized salt is represented with blue dots (68%), while the non-intake is shown with red dots (32%). Moreover, the univariate statistical analysis for a comparison between the use of iodized or non-iodized salt in the gestational period allowed us to highlight statistically significant lower levels of free carnitine (C0) in the metabolic profile of newborns from mothers who took iodized salt (*p* < 0.001), as highlighted in Figure 2C.

#### 3.1.3. Smoking

**Figure 3:**
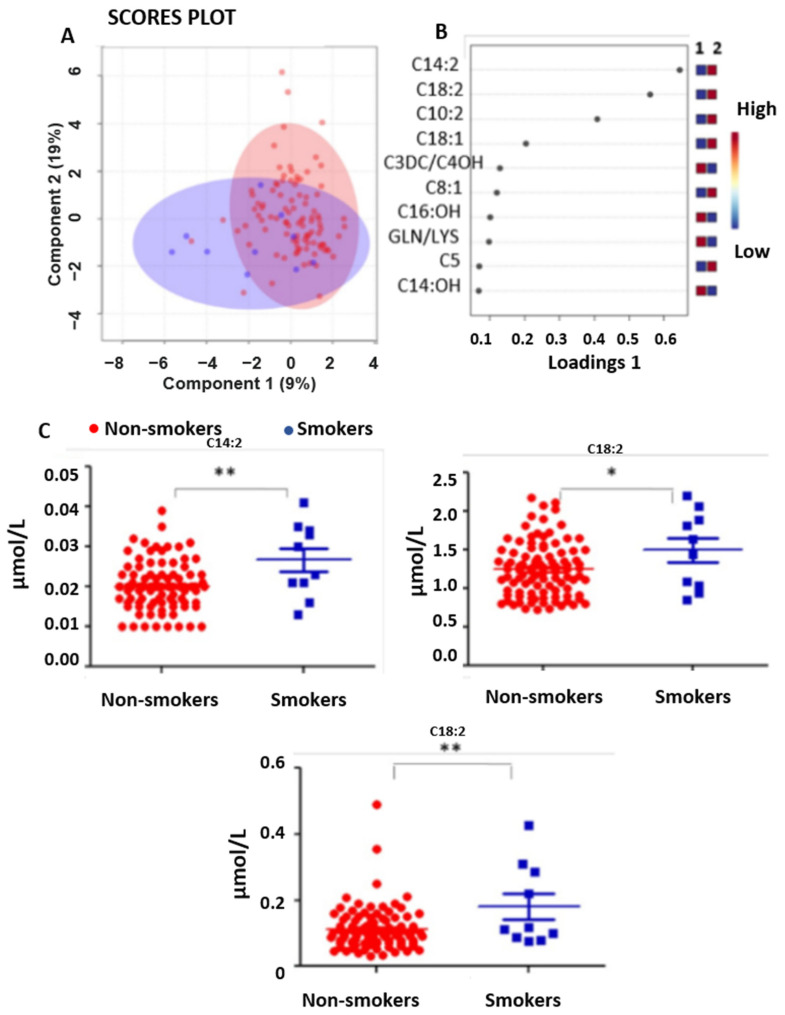
(**A**,**B**) PLS-DA scattered analysis: selection and clustering of variables relating to smoking: non-smoker mothers in red, smoker mothers in blue. (**C**) Dots plot shows the concentrations in μmol/L for C14:2, C18:1 and C18:2 in newborns with mothers without a habit of cigarette smoking (in red) and for mothers with this habit (in blue) during pregnancy. (C14:2: *p* = 0.0015, C18:1: *p* = 0.0392, and C18:2: *p* = 0.0032). (*) *p* < 0.05 and (**) *p* < 0.01.

In addition, another factor analyzed was smoking. By segregating the matrix as a function of smoking during pregnancy, the sPLS-DA statistical analysis showed a fair separation between the two groups: mothers without a habit of cigarette smoking during pregnancy in red (91%) and mothers with this habit in blue (9%), as depicted in Figure 3A,B.

Keeping in mind the statistical limitations due to the fact that the cohort of smokers was far lower than that of non-smokers, univariate statistical analysis in Figure 3 panel C pointed out some acylcarnitines, i.e., ocatadecenoylcarnitine (C18:1), octadecadienoylcarnitine (C18:2) and tetradecenoylcarnitine (C14:1), as statistically significant features (C14:2 and C18:2 *p* < 0.001; C18:1 *p* = 0.0392).

### 3.2. Cluster Analysis

**Figure 4:**
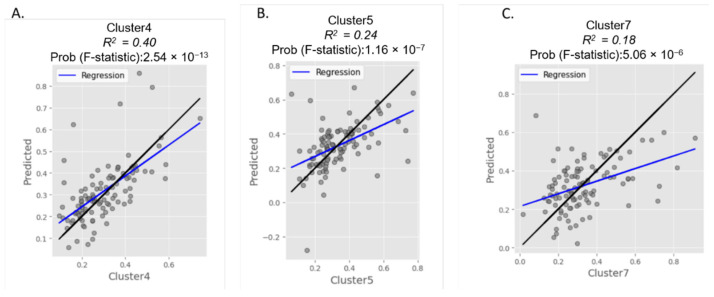
(**A**) Correlation analysis between cluster 4 (quantified odd chain acylcarnitines in newborns) and maternal responses to the questionnaire. The best features are represented by gestational age, newborn weight, and antibiotic use. (**B**) Correlation analysis between cluster 5 (quantified hydroxylated and dicarboxylic acylcarnitines in newborns) and maternal responses to the questionnaire. The best feature is represented by the frequency of taking nervine drinks during pregnancy. (**C**) Correlation analysis between cluster 7 (quantified, long, and very long chain acylcarnitines in newborns) and maternal responses to the questionnaire. The best feature is represented by the frequency of taking supplements during pregnancy.

The quantified metabolites were normalized and grouped into 7 clusters: Cluster 1 containing amino acids, Cluster 2 containing urea cycle amino acids, and metabolites, Cluster 3 containing C0, C2 and C4, Cluster 4 containing the odd acylcarnitines C3 and C5 and the C3/C2 and C3/C16 ratios, Cluster 5 containing the sum of hydroxylated and dicarboxylic acylcarnitines, Cluster 6 containing the medium chain acylcarnitines and finally Cluster 7 containing long and very long chain acylcarnitines. A correlation analysis was carried out between the groups of metabolites quantified in the DBS of newborns with some maternal food habits and lifestyles as well as with some neonatal features. The correlation analysis revealed a significant correlation between Cluster 4 of odd-chain acylcarnitines and some information relating to the newborn’s gestational age, weight, and use of antibiotics (R^2^ = 0.40 and F-statistic = 2.54 × 10^−13^) (Figure 4A). This first result demonstrated how the prematurity of the newborn affected the levels of some metabolites, such as acylcarnitine C5 and the C3/C16 ratio, resulting in a significant modulation as reported in Figure S1 of the Supplementary Materials. Cluster 5 showed a good correlation between the frequency of consumption of nervine drinks in pregnancy and the levels of hydroxylated and dicarboxylic acylcarnitines in the newborn, with R^2^ = 0.24 and F-statistic = 1.16 × 10^−7^ (Figure 4B). Finally, this correlation analysis showed that Cluster 7 of long-chain acylcarnitines was significantly correlated with the frequency of taking supplements during pregnancy, with R^2^ = 0.18 and F-statistic = 5.06 × 10^−6^ (Figure 4C).

It must be pointed out that all the metabolites analyzed, both in their absolute value and individually, were all within the reference limits used in the routine analysis for the diagnosis of related metabolic diseases. Furthermore, no significance emerged from the univariate statistical analysis except for the odd chain acylcarnitines C5 and C3/C16 ratio, which was significantly modulated in relation to gestational age but not to maternal nutrition and lifestyle.

## 4. Discussion

Many authors have already discussed how the expansion of NBS has highlighted a number of “*side effects*”, even when considering the significant advantages achieved in the diagnosis and treatment of IEMs [4,5,6,13]. Lanpher B et al., in their review, gave a significant description of the importance of metabolite flux and of the integration of phenotypic characterization with environmental, genetic, and biochemical factors. They clarified the crucial role of this integration in leading to new diagnostic and therapeutic approaches [14]. In this view, it is worth remembering that a metabolic profile is not exclusively influenced by the genome but also by a variety of clinical variables as well as external factors that may have an impact on it. Actually, it is well known that alterations in specific markers by MS/MS NBS allow for the identification of affected patients, but this may also be a consequence of maternal defects, nutritional deficiency, partial enzyme deficiency, late-onset forms of the disease, heterozygosis variants, prematurity, and the use of drugs [4,5,6].

In the present study, we investigated a possible correlation between maternal lifestyle and dietary habits during pregnancy and the newborn metabolic profile, using the data obtained by the NBS laboratory of Abruzzo Region, Italy. As regards physical activity, high concentrations of GLN in women who exercised during pregnancy suggested a mobilization of amino acids in the muscles and a probable role of GLU and GLN in the detoxification processes. At the same time, an optimized function of the immune system was also described in newborns born by physically active mothers [15]. GLU in high quantities could be used partly for GLN formation, which is essential in synthesis and immunocompetence processes and for transamination reactions. Furthermore, it could be used partly to enter the Krebs cycle as an energy substrate in the form of α-ketoglutarate [16,17].

Maternal iodized salt consumption has been associated with lower levels of neonatal C0 in our study. Iodine is necessary for thyroid hormone synthesis, and a severe deficiency causes hypothyroidism, and a higher iodine intake is often required during pregnancy [18]. Carnitine is a quaternary ammonium compound. It transports fatty acids across the mitochondrial membrane as acyl carnitine esters so that these can be metabolized in the beta-oxidation of fatty acids. Carnitine determines good cardiac function and a reduction in oxidative stress, inflammation, and necrosis processes. The relationship between carnitine and thyroid function comes from clinical studies showing the antagonistic thyroid hormone action effects of C0 [19,20,21,22] and a positive correlation between urinary carnitine with serum thyroxin concentration [23]. In our study, C0 was lower in newborns whose mothers used iodized salt during pregnancy (Figure 2C). It is challenging to explain these data, mainly because we do not have maternal TSH and thyroid hormone levels since it does not represent a routine investigation in NBS. However, we did carry out a correlation between the levels of free carnitine in the newborn with their TSH values, measured contextually after about 48 h of life, finding no correlation, as reported in Figure S2 of the Supplementary Materials. To the best of our knowledge, there are no studies evaluating the relation of the neonatal carnitines profile and maternal thyroid function. A really recent study addressed the correlation between maternal thyroid hormones, TSH, and C0 with birth weight showing a positive correlation between maternal C0, TSH, and thyroid hormones and a negative association between C0, free thyroxine, and birth weight [24]. Following this questionnaire, in our study, a significant decrease in the levels of C0 was revealed in the metabolic profile of newborns whose mothers (68% of participants) took iodized salt during pregnancy. In this context, when focusing on iodine intake, it would be also interesting to evaluate the geographical origin (sea areas or mountain ones) of mothers [25] and the condition of hypothyroidism, hyperthyroidism, or euthyroidism. As regards smoking, acylcarnitines, such as C18: 1, C18: 2, and C14: 1, were all higher in the metabolic profiling of newborns from pregnant smokers. It has been already reported that cigarette smoking causes a depletion of ascorbic acid, which is a cofactor for the enzymes trimethyl-lysine hydroxylase and gamma-butyrobetaine hydroxylase involved in the synthesis of carnitine [26,27]. From this point of view, we would expect that cigarette smoking may lead to a reduction in unsaturated carnitines and an increase in hydroxylated ones. The reversal of this trend we observed in the newborns’ metabolic profiling would make us speculate on a possible compensatory effect: an adaptive response that the fetus acquires in response to the oxidative stress induced by smoking in the mother’s body. To support and eventually confirm this hypothesis, it would be essential to use a larger cohort.

Finally, from the correlation analyses obtained by clustering the metabolites quantified in the newborns, some interesting correlations emerged, above all relating to the frequency of the intake of supplements and the frequency of consuming nervine drinks during pregnancy. As discussed above, when looking at each metabolite separately, no significance emerged, with the exception of the odd chain acylcarnitines, which are statistically related to the condition of the relative prematurity of the newborn.

Post-analytical interpretive tools, often applied by NBS programs worldwide, may resolve the issue of false-positive screens for IEMs by using the information on a variety of clinical variables potentially associated with abnormal results, such as birth weight, gestational age, gender, time at blood collection, antibiotic and/or cortisone treatment, and type of nutrition. In this way, the maternal nutritional information and lifestyle collected by our questionnaire could be helpful in the management of newborn clinical outcomes in the post-analytical stage to potentially minimize false-positive results, thus enabling a personalized medicine approach. We are aware that this must be considered as a preliminary study, based on a limited number of participants, while also considering that our region was very small and mother participation was on a voluntary basis. Nevertheless, achieving a higher number of participants over time would be necessary to provide a post-analytical tool that is suitable for supporting clinical applications in NBS laboratories.

## 5. Conclusions

In this study, by correlating the data obtained from the NBS laboratory of the Abruzzo region in Italy with the results of the questionnaire we developed, it was possible and interesting to investigate the impact of mothers’ lifestyles and dietary habits during pregnancy on newborns’ metabolic profile. Of note, physical activity, the use of iodized salt, consumption of supplements and nervine drinks, as well as smoking were all found to correlate with specific metabolites among those investigated in newborns. However, we recognize that it is important to confirm these correlations in a larger cohort and be able to achieve a global and complete knowledge of newborns and mothers. In doing so, it would be possible to reconsider the newborn metabolic profile by expanding NBS in view of the maternal lifestyle and dietary habits for an improved understanding and a better diagnostic evaluation.

## Data Availability

The data presented in this study are available in Supplementary Materials.

## Supplementary Materials

The following supporting information can be downloaded at: https://www.mdpi.com/article/10.3390/nu15102297/s1, Figure S1. C5 and C3/C16 ratio univariate statistical analysis performed by Metaboanalyst based on data from Cluster 3, considering gestational week (g.w.) and antibiotics treatment in newborn. Figure S2. Pearson correlation between DBS TSH levels (µU/mL) and DBS free carnitine C0 levels (µM). R squared is 0.00011, *p*-value 0.91 (not significant). Table S1. Mass spectrometry parameters used for FIA-MS/MS analysis on RenataDXTM Screening Systems by NeoBase™ 2 Non-derivatized MSMS kit. For each analyte, MRM transition, cone voltage (V) and collision energy (eV) are shown. The internal standards (ISs) are reported in bold. Table S2. Questionnaire.
