# Supplementary material for: Impact of Maternal Lifestyle and Dietary Habits during Pregnancy on Newborn Metabolic Profile
Source: Nutrients. 2023 May 13;15(10):2297. doi: 10.3390/nu15102297 (PMC10221063; doi:10.3390/nu15102297)
Supplement: Supplementary file 1 [file nutrients-15-02297-s001.zip › nutrients-2377744-supplementary.pdf]

Supplementary Material of:

Impact of Maternal Lifestyle and Dietary Habits during Pregnancy on Newborn Metabolic Profile

Ilaria Cicalini [1,2], Samanta Moffa [3], Maria Lucia Tommolini [1,2], Silvia Valentinuzzi [1,3], Mirco Zucchelli [1,2], Ines Bucci [1,4], Piero Chiacchiaretta [1,2], Antonella Fontana [3], Luca Federici [1,2], Vincenzo De Laurenzi [1,2], Piero Del Boccio [1,3], Claudia Rossi [1,2,*] and Damiana Pieragostino [1,2]

1 Center for Advanced Studies and Technology (CAST), "G. d'Annunzio" University of Chieti-Pescara, 66100 Chieti, Italy; ilaria.cicalini@unich.it (I.C.); maria.tommolini@unich.it (M.L.T.); silvia.valentinuzzi@unich.it (S.V.); m.zucchelli@unich.it (M.Z.); ines.bucci@unich.it (I.B.); p.chiacchiaretta@unich.it (P.C.); luca.federici@unich.it (L.F.); vincenzo.delaurenzi@unich.it (V.D.L.); p.delboccio@unich.it (P.D.B.); damiana.pieragostino@unich.it (D.P.)

2 Department of Innovative Technologies in Medicine and Dentistry, "G. d'Annunzio" University of Chieti-Pescara, 66100 Chieti, Italy

3 Department of Pharmacy, "G. d'Annunzio" University of Chieti-Pescara, 66100 Chieti, Italy; samanta.moffa@unich.it (S.M.); antonella.fontana@unich.it (A.F.)

4 Department of Medicine and Aging Science, "G. d'Annunzio" University of Chieti-Pescara, 66100 Chieti, Italy

* Correspondence: claudia.rossi@unich.it; Tel.: +39-0871-541290

Figure S1. C5 and C3/C16 ratio univariate statistical analysis performed by Metaboanalyst based on data from Cluster 3, considering gestational week (g.w.) and antibiotics treatment in newborn.

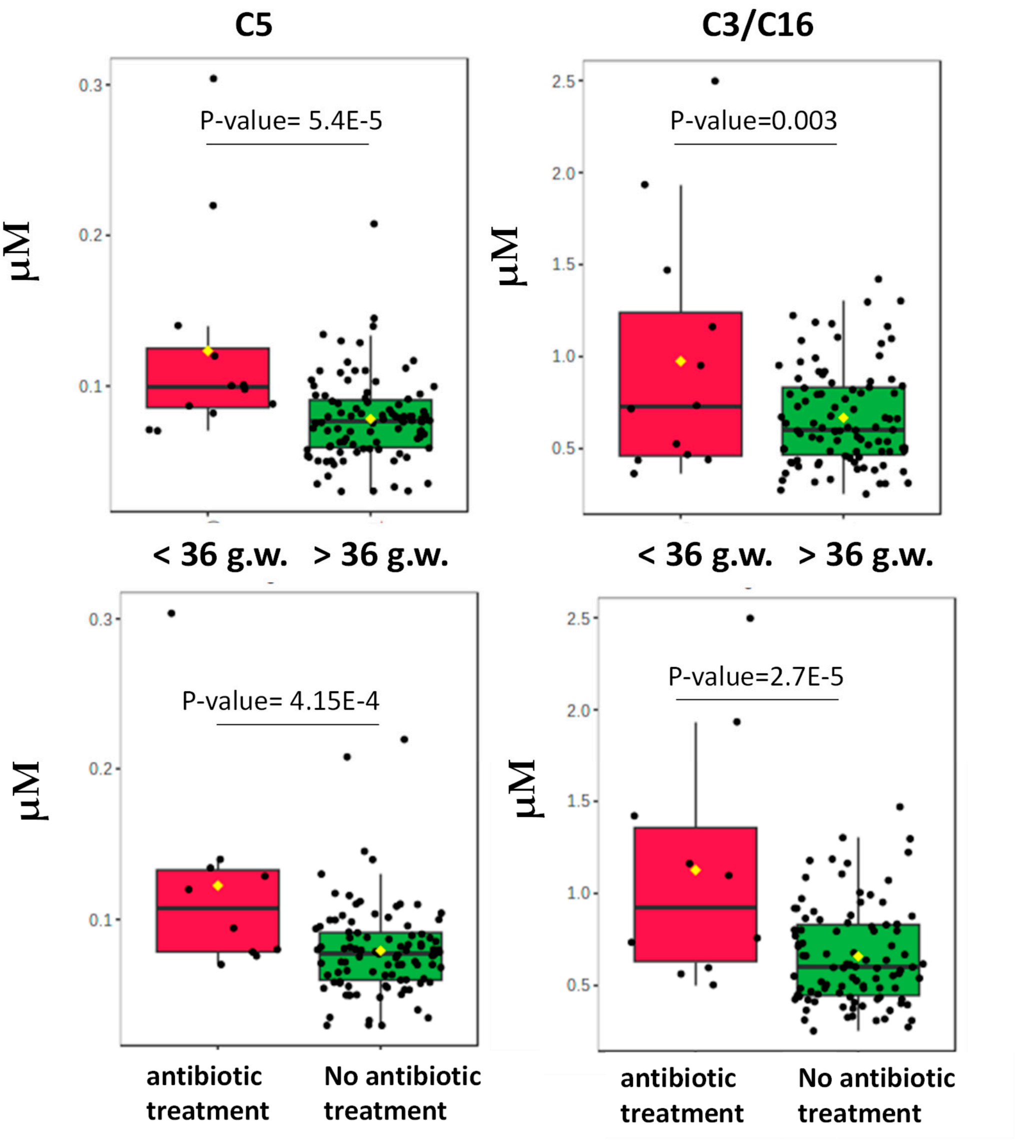

Figure S2. Pearson correlation between DBS TSH levels (μU/mL) and DBS free carnitine C0 levels (μM). R squared is 0.00011, p-value 0.91 (not significant).

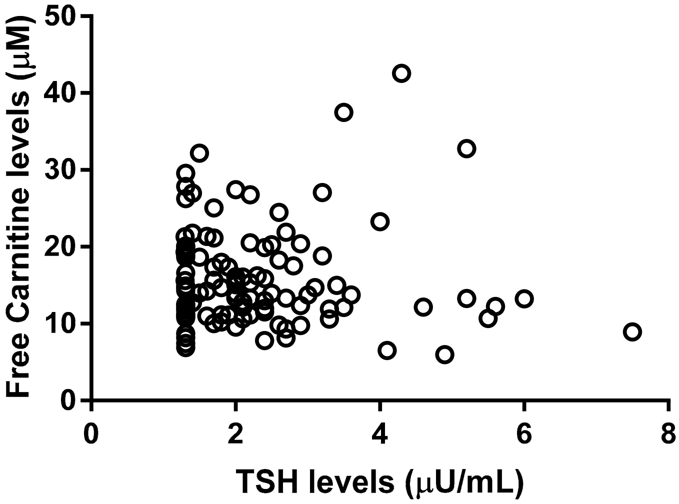

Table S1. Mass spectrometry parameters used for FIA-MS/MS analysis on RenataDX™ Screening Systems by NeoBase™ 2 Non-derivatized MSMS kit. For each analyte, MRM transition, cone voltage (V) and collision energy (eV) are shown. The internal standards (ISs) are reported in bold.

Abbreviations of analytes and ISs	Analyte	Transition	Cone potential	Collision energy
Arg<br>**$^{2}H_4$, ^{13}C-Arg**	Arginine	175.1>70.1<br>180.1>75.1	34	21
Cit<br>**2H_2-Cit**	Citrulline	176.1>113.1<br>178.1>115.1	24	16
Leu/Ile/Pro-OH<br>**$^{2}H_3$-Leu**	Leucine/Isoleucine/Hydroxyproline	132.1>86.1<br>135.1>89.1	24	10
Met<br>**$^{2}H_3$-Met**	Methionine	150.1>104.1<br>153.1>107.1	24	10
Phe<br>**$^{13}C_6$-Phe**	Phenylalanine	166.1>120.1<br>172.1>126.1	25	12
Tyr<br>**$^{13}C_6$-Tyr**	Tyrosine	182.1>136.1<br>188.1>142.1	26	12
Val<br>**$^{15}N_2$-$^{13}C_5$-Val**	Valine	118.1>72.1<br>124.1>77.1	23	10
C0<br>**$^{2}H_9$-C0**	Free carnitine	162.1>1030<br>171.2>103.0	38	16
C2<br>**$^{2}H_3$-C0**	Acetylcarnitine	204.1>85.0<br>207.1>85.0	34	18
C3<br>**$^{2}H_3$-C3**	Propionylcarnitine	218.1>85.0<br>221.2>85.0	32	18
C4<br>**$^{2}H_3$-C4**	Butyrylcarnitine	232.2>85.0<br>235.2>85.0	36	18
C5<br>**$^{2}H_9$-C5**	Valerylcarnitine	246.2>85.0<br>255.2>85.0	38	20
C6<br>**$^{2}H_3$-C6**	Hexanoylcarnitine	260.2>85.0<br>263.2>85.0	37	20
C5DC/C6OH<br>**$^{2}H_6$-C5DC**	Glutarylcarnitine/3-Hydroxy-hexanoylcarnitine	276.2>85.0<br>282.2>85.0	40	24
C8<br>**$^{2}H_3$-C8**	Octanoylcarnitine	288.2>85.0<br>291.2>85.0	42	22
C10<br>**$^{2}H_3$-C10**	Decanoylcarnitine	316.2>85.0<br>319.3>85.0	45	22
C12<br>**$^{2}H_3$-C12**	Dodecanoylcarnitine	344.3>85.0<br>347.3>85.0	46	24
C14<br>**$^{2}H_3$-C14**	Tetradecanoylcarnitine (myristoylcarnitine)	372.3>85.0<br>375.3>85.0	52	25
C16<br>**$^{2}H_3$-C16**	Hexadecanoylcarnitine (palmitoylcarnitine)	400.3>85.0<br>403.4>85.0	55	26
C18<br>**$^{2}H_3$-C18**	Octadecanoylcarnitine (stearoylcarnitine)	428.4>85.0<br>431.4>85.2	56	28

Table S2. Questionnaire

QUESTION	ANSWER (LEGEND)
**Nationality**	Italian (0)
	Albanian (1)
	Brazilian (2)
	Spain (3)
	Romanian (4)
**Age**	
**Height (cm)**	
**How much weight did you gain during pregnancy? (kg)**	
**Pre-pregnancy weight (kg)**	
**Is this your first pregnancy?**	Yes (0)
	No (1)
**Have you had abortions?**	Yes (0)
	No (1)
**Did you have toxoplasmosis before pregnancy?**	Yes (0)
	No (1)
**Do you have any of the following or another condition?**	NONE (0)
	Hypothyroidism and pathologies (1)
	Gestational diabetes (2)
	Overweight or obesity (3)
	Coagulation factor 11 deficiency (4)
	Anemia (5)
	Hypercholesterolemia (6)
**Do you have any intolerance?**	Yes (0)
	No (1)
**Specify intolerances**	NONE (0)
	Nickel lactose (1)
	Lactose, gluten (2)
	Lactose (3)
	Cheeses (4)
	Gluten (5)

	Egg (6)
	Legumes (7)
	Medicines (8)
**Do you have any allergies?**	Yes (0)
	No (1)
**Specify allergies**	None (0)
	Formaldehyde, potassium dichromate, nickel (1)
	Shellfish (2)
	Pollen (3)
	Allergic rhinoconjunctivitis (grasses) (4)
	dust, cat fur, olive on the nose (5)
	Pollen, dust (6)
	Mites (7)
	Nickel scent parietal pollen (8)
	Mold (Alternaria alternata) (9)
	Hazelnuts, peanuts (10)
	Allergic reaction to cortisone (11)
	Pollen, cat (12)
	Mushrooms, dust, grasses (13)
	Pollen, penicillin (14)
	Peanuts and soy (15)
	Azithromycin powder (16)
	Fluoroquinolones (17)
	Medicines (18)
	Nickel (19)
	Penicillin (20)
	Clams (21)
**Did you smoke?**	Yes (0)
	No (1)
**Have you done any physical activity (walking, swimming, yoga, exercise bike...)?**	Yes (0)
	No (1)

**Have you taken any medications?**	Yes (0)
	No (1)
**Specify which medications you have taken**	Nobody
	Tyrosint - Eutirox
	Cardioaspirin, tyrosint 25
	Aspirin
	Folic acid, cardioaspirin, nuperal (antinausea)
	Lentogest (progestogens)
	Anti nausea
	Antibiotic
	Tardifer (antianemic)
	Clexane 4000 -fluxum (anticoagulants)
	EPITARAM (antiepileptics)
	antibiotics + tachypirine
	Eutirox progesterone spasmex
	Tachipirina and anti-inflammatories
	Progestin and anticoagulant
	Antibiotics + cortisone
	Antidepressants
	Antibiotics + progestogens
	Cardioaspirin + progestogens
**What type of diet did you follow?**	None
	Mediterranean diet
	Gestational diabetes diet
**Did you use iodized salt?**	Yes (0)
	No (1)
**Have you used food supplements?**	Yes, occasionally
	Yes, every day
	No
**What kind of supplements did you take?**	Folic acid (0)
	Iron, Omega-3 (1)
	Folic acid, Vitamin D, iron (2)

	Folic acid, Iron (3)
	Salivit (4)
	Folic acid, Multivitamin (5)
	Folic acid, Iron, Probiotics (6)
	Nobody (7)
	Folic acid, Vitamin C (8)
	Iron (9)
	Folic acid, Vitamin B12, Iron, Magnesium (10)
	Folic acid, Vitamin K, Calcium, Magnesium (11)
	Also used after childbirth:
	Vitamin D (13)
	Magnesium (14)
**Are you taking any medications?**	Yes (0)
	No (1)
**Specify which medications you are taking**	Nobody (0)
	Tyrosint - Eutirox (1)
	Epitaram (2)
	Sereupin - Patoxetine (antidepressants) (3)
	Tardifer (4)
	To regulate blood pressure (5)
	Antibiotics (6)
**Are you using dietary supplements?**	Yes, occasionally (0)
	Yes, every day (1)
	No (2)
**Toxoplasmosis test during pregnancy**	Positive (0)
	Negative (1)
**Do you prefer whole grains?**	Yes (0)
	No (1)
**What condiments do you prefer?**	Extra virgin olive oil (0)
	Extra virgin olive oil, Butter (1)
	Extra virgin olive oil, seed oil, butter (2)

	Extra virgin olive oil, butter, ghee (3)
	Extra virgin olive oil, Seed oil (4)
**What type of diet do you currently follow?**	None (0)
	Mediterranean diet (1)
	Ketogenic (2)
**Your baby is fed with**	Mother's milk (0)
	Breast milk, Artificial milk (1)
	Artificial milk (2)
	Special milk (3)
**What type of diet did you follow?**	None (0)
	Mediterranean diet (1)
	Gestational diabetes diet (2)
